# A rare pediatric case of McCune–Albright syndrome with acute visual disturbance: Case report

**DOI:** 10.1097/MD.0000000000028815

**Published:** 2022-02-11

**Authors:** Hiroshi Ninomiya, Michio Ozeki, Akifumi Nozawa, Shiho Yasue, Saori Endo, Masayuki Inuzuka, Natsuko Obara, Kiyofumi Mochizuki, Masaya Kawaguchi, Yo Kaneko, Naoyuki Ohe, Yoko Aoki, Masayuki Matsuo, Toru Iwama, Hidenori Ohnishi

**Affiliations:** a Department of Pediatrics, Graduate School of Medicine, Gifu University, Yanagido, Gifu, Gifu, Japan; b Department of Medical Genetics, Tohoku University School of Medicine, Sendai, Japan; c Department of Ophthalmology, Graduate School of Medicine, Gifu University, Yanagido, Gifu, Gifu, Japan; d Department of Otorhinolaryngology, Graduate School of Medicine, Gifu University, Yanagido, Gifu, Gifu, Japan; e Department of Radiology, Graduate School of Medicine, Gifu University, Yanagido, Gifu, Gifu, Japan; f Department of Neurosurgery, Graduate School of Medicine, Gifu University, Yanagido, Gifu, Gifu, Japan.

**Keywords:** aneurysmal bone cyst, case report, fibrous dysplasia, McCune–Albright syndrome, orbital apex syndrome

## Abstract

**Rationale:**

McCune–Albright syndrome (MAS) is a rare disorder characterized by clinical findings, which includes fibrous dysplasia (FD). FD is a benign tumor that leads to increased rates of bone fracture. In some MAS cases with FD, facial deformities, severe pain, and orbital neuropathies are complicated. Aneurysmal bone cyst (ABC) is a benign bone tumor and rare complication of FD.

**Patient concerns:**

A 9-year-old boy was admitted to our hospital because of acute visual disturbance.

**Diagnosis and interventions:**

The patient was clinically diagnosed as ABC complicated with MAS, and he underwent surgery.

**Outcomes:**

After the surgery, his sight became normal. Recurrence of ABC and visual disturbance was not observed in 3 years. Genetic analysis of a tissue sample from the ABC lesion by next-generation sequencing revealed a somatic activating *GNAS* mutation.

**Lessons:**

To the best of our knowledge, this is the first case report of MAS causing optic neuropathy complicated with ABC. ABC complicated with MAS is extremely rare, but it should be considered as a possible diagnosis in patients with acute visual loss and facial swelling. In addition, our case had OAS, which is an uncommon syndrome and a rare complication in ABC with MAS, and rapid decompression of the ABC was effective in improving the patient's eyesight.

## 1 Introduction

McCune–Albright syndrome (MAS) is a rare disease in which the major symptoms are endocrine disorder, café-au-lait pigmentation, and fibrous dysplasia (FD). The estimated prevalence of MAS is between 1/100,000 and 1/1,000,000.^[1]^ A somatic mutation in *GNAS* gene that activates the Gs protein α subunit is known to cause the pathogenesis of MAS. FD is a benign tumor that leads to increased rates of bone fracture. In some MAS cases with FD, facial deformities, severe pain, and orbital neuropathies are complicated.^[2]^

Aneurysmal bone cysts (ABCs) are blood-filled cysts that often occur as primary lesions. The etiology of ABC is unclear, and secondary ABC co-existing with other legions such as giant cell tumor and chondromyxoid fibroma accounts for 30% of whole ABC.^[3]^ ABC is a rare complication of FD, and only 2 cases of ABC complicated with FD in MAS were previously reported.^[4,5]^

We report an extremely rare case of ABC in a MAS patient who presented acute visual disturbance and who was successfully treated by percutaneous surgery and steroids.

## 2 Case presentation

**Figure 1:**
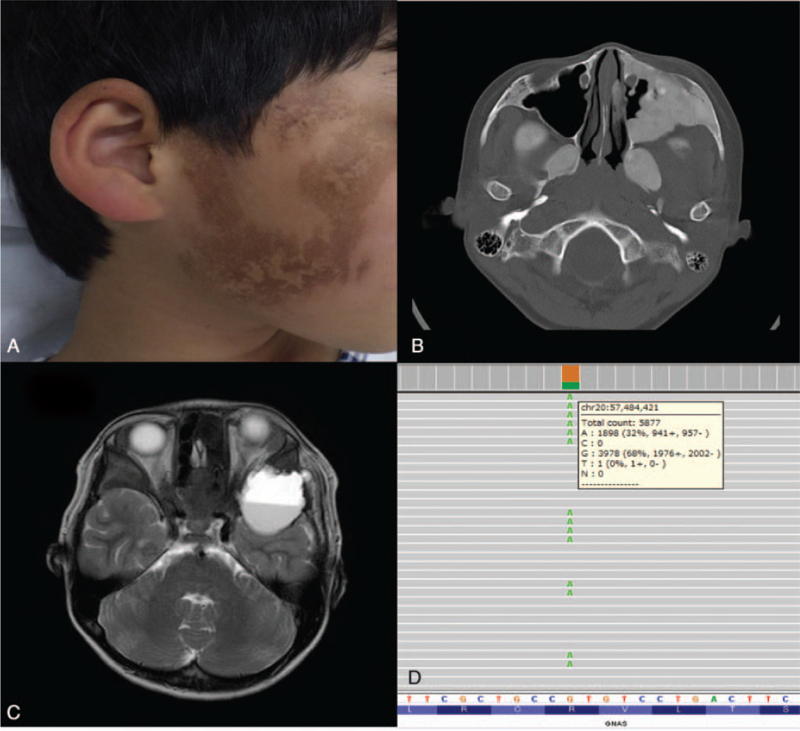
Dermatological findings and radiological examination of a patient with fibrous dysplasia and aneurysmal bone cyst before treatment and genetic analysis. (A) Café-au-lait macules from birth on the right-hand side of the patient's face were identified. (B) CT revealed expansile ground-glass opacity on his bilateral pterygoid process and on the posterior wall of the left maxillary sinus (axial CT bone window of the skull base showed ground-glass opacification on his bilateral pterygoid processes and on the anterior and posterior bony walls of the maxillary sinus) and we diagnosed fibrous dysplasia. (C) Transverse T2-weighted MRI showed the cystic bone tumor with fluid-fluid level in the left middle cranial fossa with left optic nerve compression, indicating secondary ABC. (D) Genetic analysis by next-generation sequencing showed a somatic activating mutation in *GNAS* at c.602G > A (p.R201H) at a variant allele frequency of 32.3%. ABC = aneurysmal bone cyst, CT = computed tomography, MRI = magnetic resonance imaging.

A 9-year-old boy had ophthalmalgia for 1 month, with diplopia and exophthalmos of the left eye that emerged later. His previous medical and family history was unremarkable. He visited our hospital for further evaluation and treatment. Physical examination revealed his height and weight were appropriate for his age, and café-au-lait macules were evident on the right-hand side of his face (Fig. 1A). Ocular movements of the left eye were restricted in all directions. The best corrected visual acuity was 12/20 in the left eye. No other abnormalities in the function of his cranial nerves were identified in funduscopic examination. Laboratory tests including endocrinological examination did not reveal any abnormalities. Computed tomography (CT) on the first day showed multiple FD in his sphenoid bone, left maxillary bone, and right femur, and a cyst in his left temporal bone (Fig. 1B). T2-weighted magnetic resonance imaging (MRI) on the third day revealed fluid-fluid levels on his middle cranial fossa with left optic nerve compression (Fig. 1C). These clinical and radiological findings were matched to the MAS and FD from ABC. A test for relative afferent pupillary defect (RAPD) was positive in the left eye, and ptosis in the left eye was observed. These symptoms were considered to be caused by the compression of the ABC and matched the diagnosis of orbital apex syndrome (OAS). We started treatment with glucocorticoids, and then performed a partial resection of the dysplasia and opened the cyst component to decompress the left optic nerve on the fourth day. The pathological features of the removed lesion were consistent with FD. After his surgery, his pupillary light reflex improved in 4 days and the left eye also gradually opened. His visual acuity in the left eye returned to normal at the end of 3 months. Since his discharge, recurrence of ABC and visual disturbance has not been observed in 3 years.

We conducted genetic tests to confirm the diagnosis after his discharge. Genetic analysis of a tissue sample from the ABC lesion by next-generation sequencing was performed using a targeted 50-gene panel (AmpliSeq for Illumina Cancer Hotspot Panel v2; Illumina, Hayward, CA) that includes *GNAS*. A known somatic activating mutation in *GNAS*, c.602G > A (p.R201H), was detected as a variant allele at a frequency of 32.3% in the ABC lesion (Fig. 1D). Following the identification of these clinical features and the genetic mutation, his diagnosis was confirmed.

## 3 Discussion

MAS is caused by a somatic *GNAS* mutation that can lead to various characteristic symptoms of MAS. Genetic testing is generally useful for confirming a diagnosis of MAS; however, it is often diagnosed by its typical clinical findings.^[1]^ In our case, the optic nerve disorder resulted in the selection of early surgery to prevent sequelae.

FD is the most common symptom in MAS and is observed in 98% of MAS patients.^[6]^ The pathophysiological process of FD involves the replacement of normal bone by immature myeloid stromal cells in which differentiation has been stopped because of a somatic mutation in *GNAS*.^[7]^ Therefore, *GNAS* causes FD as in MAS.^[8]^ There are 2 types of ABC: primary ABC, which is characterized by a *USP6* or *CDH11* gene rearrangement^[9]^; and secondary ABC, which may arise as a reactive process in association with almost any other benign or, less commonly, malignant bone tumor.^[10]^ Secondary ABC does not harbor a *USP6* or *CDH11* alteration, although the detection of a genetic aberration characteristic of the primary tumor, such as *GNAS* R201 alterations in FD, can assist in a diagnosis.^[11]^ Secondary ABC is a morphological mimic of primary ABC but lacks the hallmark *USP6* or *CDH11* rearrangements of primary ABC, and possibly has varied genetic features corresponding to the specific bone tumors.^[10]^ In FD patients, craniofacial lesions are noted in 90% of cases. Furthermore, 95% of FD patients with craniofacial lesions have skull base lesions and 70% of cases have temporal bone lesions.^[12]^ Although 50% to 90% of FD cases have the radiological features of optic nerve compression,^[12]^ most cases are asymptomatic.^[7]^ Moreover, Couturier et al^[13]^ reported that optic neuropathy occurring in 10% of patients with FD presented radiological features of optic nerve compression. The reported causes of optic neuropathy in FD patients are optic neuropathy caused by central retinal artery occlusion or papillary edema associated with increased intracranial pressure, optic nerve compression with hemorrhage in FD, and calcification of the optic nerve tube with ABC.^[14]^ In our case, the acute visual disturbance caused by optic neuropathy was due to compression of the optic canal by ABC enlargement with bleeding. Orbital ABC is rare but should be considered as a diagnosis.

ABC is also a benign tumor and often occurs in long bones such as the femur,^[15]^ but orbital lesions are rare and are observed in less than 0.25% of cases.^[16]^ MRI reveals the apparently circumscribed, expansile, and lytic lesions of typical ABC.^[3]^ The interiors of ABC lesions are often filled with amber liquid^[16]^; therefore, the diagnosis of ABC is often made upon the observation of a fluid-fluid level on CT or MRI.^[15]^ Thirty percent of ABC are secondary changes and are derived from chondroblastoma and giant cell tumors, and rarely from FD. In addition, secondary changes of ABC in MAS complicated with FD are exceptional, and only 2 cases have been reported. The first reported case was in a 14-year-old girl^[5]^ whose lesion was present in the bilateral occipital bones, and the second case was a 28-year-old woman^[4]^ who had a pelvic lesion (Table 1). The 14-year-old girl was treated with resection and the 28-year-old woman did not agree to treatment. Earlier glucocorticoid treatment and percutaneous decompression could have led to an improvement in orbital neuropathy and eye prognosis.

**Table 1:** The clinical features of aneurysmal bone cyst complicated with McCune-Albright syndrome.

Age/gender	Symptoms	Location	Treatment	References
28/F	Pain, bone enlarging	Pelvic	No	Tournis et al^[4]^
14/F	Bone enlarging	Occipital bone	Resection	Urgun et al^[5]^
9/M	Pain, diplopia, ophthalmalgia	Temporal bone	glucocorticoids and percutaneous decompression	

F = female, M = male.

In our case, the patient presented OAS in the acute phase. OAS defined by Kjoer shows oculomotor cranial nerve injury associated with optic nerve dysfunction.^[17]^ OAS results from neoplastic, inflammatory, and vascular conditions. The ABC disturbed the II, III, IV, and V cranial nerves in our patient. Surgical resection, radiation therapy, and chemotherapy should be considered as the treatment for OAS caused by neoplasm. We treated our patient with percutaneous decompression. OAS is uncommon and, to our knowledge, no case of OAS complicated with ABC and MAS has been reported. However, there is no definitive treatment for MAS with optic neuropathy, and the standard treatment for ABC is resection. In view of the patient's visual prognosis, surgical treatment should be selected for MAS patients with orbital neuropathy because it can quickly eliminate the cause.

## 4 Conclusion

FD sometimes causes orbital neuropathy and poor eye prognosis; however, the treatment for FD is not clear. ABC complicated with MAS is extremely rare, but it should be considered as a possible diagnosis in patients with acute visual loss and facial swelling. In addition, our case had OAS, which is an uncommon syndrome and a rare complication in ABC with MAS, and rapid decompression of the ABC was effective in improving the patient's eyesight.

## Acknowledgments

We are grateful to the patients and their families for their invaluable contributions to this study. We thank the members of the Department of Pediatrics of Gifu University and Professor Toshiyuki Fukao for their contributions. We thank Dr Koki Nagai, Dr Taiki Abe, Ms Yoko Tateda, and Dr Tetsuya Niihori of Tohoku University for providing technical assistance with the next-generation analysis. We also acknowledge the technical support of the Biomedical Research Core of Tohoku University Graduate School of Medicine. Finally, we thank H. Nikki March, PhD, from Edanz Group (https://en-author-services.edanz.com/ac) for editing a draft of this manuscript.

## Author contributions

**Conceptualization:** Hiroshi Ninomiya, Michio Ozeki, Hidenori Ohnishi.

**Data curation:** Hiroshi Ninomiya, Michio Ozeki, Akifumi Nozawa, Shiho Yasue, Saori Endo, Masayuki Inuzuka, Natsuko Obara, Kiyofumi Mochizuki, Masaya Kawaguchi, Yo Kaneko, Naoyuki Ohe, Yoko Aoki, Masayuki Matsuo, Toru Iwama, Hidenori Ohnishi.

**Formal analysis:** Michio Ozeki, Akifumi Nozawa, Shiho Yasue, Saori Endo, Masayuki Inuzuka, Natsuko Obara, Kiyofumi Mochizuki, Masaya Kawaguchi, Yo Kaneko, Naoyuki Ohe, Yoko Aoki, Masayuki Matsuo, Toru Iwama, Hidenori Ohnishi.

**Funding acquisition:** Michio Ozeki, Yoko Aoki.

**Investigation:** Hiroshi Ninomiya, Michio Ozeki, Akifumi Nozawa, Shiho Yasue, Masayuki Inuzuka, Natsuko Obara, Naoyuki Ohe, Toru Iwama, Hidenori Ohnishi.

**Methodology:** Michio Ozeki, Akifumi Nozawa, Shiho Yasue, Masaya Kawaguchi, Naoyuki Ohe, Hidenori Ohnishi.

**Project administration:** Michio Ozeki.

**Writing – original draft:** Hiroshi Ninomiya, Michio Ozeki, Hidenori Ohnishi.

**Writing – review & editing:** Hiroshi Ninomiya, Michio Ozeki, Akifumi Nozawa, Shiho Yasue, Saori Endo, Masayuki Inuzuka, Natsuko Obara, Kiyofumi Mochizuki, Masaya Kawaguchi, Yo Kaneko, Naoyuki Ohe, Yoko Aoki, Masayuki Matsuo, Toru Iwama, Hidenori Ohnishi.

## References

[1] Dumitrescu CE Collins MT. McCune-Albright syndrome. Orphanet J Rare Dis 2008;3:12. 18489744 10.1186/1750-1172-3-12 PMC2459161

[2] Wang Y Wang O Jiang Y. Efficacy and safety of bisphosphonate therapy in mccune-albright syndrome-related polyostotic fibrous dysplasia: a single-center experience. Endocr Pract 2019;25:23–30. 30383490 10.4158/EP-2018-0328

[3] Mascard E Gomez-Brouchet A Lambot K. Bone cysts: unicameral and aneurysmal bone cyst. Orthop Traumatol Surg Res 2015;101:S119–127. 25579825 10.1016/j.otsr.2014.06.031

[4] Tournis S Balanika A Megaloikonomos PD. Secondary aneurysmal bone cyst in McCune-Albright syndrome. Clin Cases Miner Bone Metab 2017;14:332–5. 29354163 10.11138/ccmbm/2017.14.3.332 PMC5762225

[5] Urgun K Yilmaz B Toktaş ZO. Craniospinal polyostotic fibrous dysplasia, aneurysmal bone cysts, and Chiari type 1 malformation coexistence in a patient with McCune-Albright syndrome. Pediatr Neurosurg 2016;51:253–6. 27161212 10.1159/000444937

[6] Collins MT Singer FR Eugster E. McCune-Albright syndrome and the extraskeletal manifestations of fibrous dysplasia. Orphanet J Rare Dis 2012;7:S4. 22640971 10.1186/1750-1172-7-S1-S4 PMC3359955

[7] Amit M Collins MT FitzGibbon EJ, et al. Surgery versus watchful waiting in patients with craniofacial fibrous dysplasia: a meta-analysis. PLoS One 2011;6:e25179. 21966448 10.1371/journal.pone.0025179 PMC3179490

[8] Weinstein LS Shenker A Gejman PV, et al. Activating mutations of the stimulatory G protein in the McCune-Albright syndrome. N Engl J Med 1991;325:1688–95. 1944469 10.1056/NEJM199112123252403

[9] Oliveira AM Hsi BL Weremowicz S. USP6 (Tre2) fusion oncogenes in aneurysmal bone cyst. Cancer Res 2004;64:1920–3. 15026324 10.1158/0008-5472.can-03-2827

[10] Oliveira AM Perez-Atayde AR Inwards CY. USP6 and CDH11 oncogenes identify the neoplastic cell in primary aneurysmal bone cysts and are absent in so-called secondary aneurysmal bone cysts. Am J Pathol 2004;165:1773–80. 15509545 10.1016/S0002-9440(10)63432-3 PMC3278819

[11] Idowu BD Al-Adnani M O’Donnell P. A sensitive mutation-specific screening technique for GNAS1 mutations in cases of fibrous dysplasia: the first report of a codon 227 mutation in bone. Histopathology 2007;50:691–704. 17493233 10.1111/j.1365-2559.2007.02676.x

[12] Lee JS FitzGibbon E Butman JA. Normal vision despite narrowing of the optic canal in fibrous dysplasia. N Engl J Med 2002;347:1670–6. 12444181 10.1056/NEJMoa020742

[13] Couturier A Aumaître O Gilain L, et al. Craniofacial fibrous dysplasia: a 10-case series. Eur Ann Otorhinolaryngol Head Neck Dis 2017;134:229–35. 28302454 10.1016/j.anorl.2017.02.004

[14] Katz BJ Nerad JA. Ophthalmic manifestations of fibrous dysplasia: a disease of children and adults. Ophthalmology 1998;105:2207–15. 9855148 10.1016/S0161-6420(98)91217-9

[15] Park HY Yang SK Sheppard WL. Current management of aneurysmal bone cysts. Curr Rev Musculoskelet Med 2016;9:435–44. 27778155 10.1007/s12178-016-9371-6 PMC5127951

[16] Menon J Brosnahan DM Jellinek DA. Aneurysmal bone cyst of the orbit: a case report and review of literature. Eye (Lond) 1999;13:764–8. 10707141 10.1038/eye.1999.224

[17] Kjœr I. A case of orbital apex syndrome in collateral pansinusitis. Acta Ophthalmol 1945;23:357–66.

